# A pan-cancer analysis of the oncogenic role of YKT6 in human tumors

**DOI:** 10.1097/MD.0000000000033546

**Published:** 2023-04-14

**Authors:** Xuezhong Zhang, Mark Lloyd G. Dapar, Xin Zhang, Yingjun Chen

**Affiliations:** a Department of Biology, College of Arts and Sciences, Central Mindanao University, Musuan, Philippines; b Department of Laboratory Medicine, Zibo Central Hospital, Zibo, China; c Center for Biodiversity Research and Extension in Mindanao, Central Mindanao University, Musuan, Philippines; d Microtechnique and Systematics Laboratory, Natural Science Research Center, Musuan, Philippines; e Department of Infectious Diseases, Binzhou Medical University Hospital, Binzhou, China.

**Keywords:** analysis, pan-cancer, prognosis, tumor, YKT6

## Abstract

YKT6, as a Soluble N-ethylmaleimide-sensitive factor attachment protein receptor (SNARE) protein with vesicle trafficking, plays an essential role in the development and progression of tumor. However, the gene of YKT6 has not been fully assessed in pan-cancer studies. We aim to investigate the gene of YKT6 across 33 different types of tumor by using the Cancer Genome Atlas, Gene Expression Omnibus database, and other several kinds of bioinformatic tools. YKT6 is significantly up-regulated in most tumors, and we found that overexpression of YKT6 is positively associated with poor prognosis of overall survival and poor disease-free survival prognosis in several tumors, such as Adrenocortical carcinoma, Bladder Urothelial Carcinoma, Head and Neck squamous cell carcinoma. We also detected distinct associations exist between YKT6 and tumor mutational burden or microsatellite instability with tumors. YKT6 expression was positively related to cancer-associated fibroblasts for TCGA tumors of colon adenocarcinoma and LGG. Furthermore, we discovered a significantly positively correlation between YKT6 expression and endothelial cell in tumors of colon adenocarcinoma, HNSC-HPV+, OV, READ and THCA. While a negative relationship was obtained between YKT6 expression and endothelial cell in KIRC. Moreover, “Syntaxin binding,” “SNARE complex,” “vesicle fusion” and “DNA replication” are involved in the influence of YKT6 on tumor pathogenesis. Our pan-cancer analysis offers a deep comprehending the gene of YKT6 in tumoeigenesis from viewpoint of clinical tumor samples.

## 1. Introduction

Due to the complexity of tumorigenesis, it is significant to perform pan-cancer gene expression analyses and explore the association between clinical prognosis and possible molecular mechanisms.^[1]^ Both publicly funded the Cancer Genome Atlas (TCGA) and Gene Expression Omnibus (GEO) database provide functional genomics data about a wide variety of tumors and enable us to conduct pan-cancer analyses.

Soluble N-ethylmaleimide-sensitive factor attachment protein receptors (SNAREs) proteins are commonly responsible for helping vesicle trafficking between membranes.^[2,3]^ It is the main fusion of SNAREs to mediate membrane fusion.^[4,5]^ SNAREs possess membrane specificity, and they undergo multiple posttranscriptional modifications that might regulate their function. Our research is focused on YKT6, as a SNARE protein with vesicle trafficking, was initially discovered in yeast.^[6]^ It has been demonstrated that YKT6 is involved in trafficking of membrane vesicles both inside and outside of the Golgi.^[7]^ Recently researches showed that YKT6 played an essential role in the development and progression of tumor. It has been found that YKT6 was required for exosome secretion and adversely impacted prognosis of non-small-cell lung cancer (NSCLC) patients.^[8,9]^ Researchers also found that YKT6 was upregulated in breast cancer samples with an invasive phenotype, promoting cell proliferation and conferring drug resistance.^[10]^ Hepatocellular carcinoma (HCC) study has revealed that overexpressed expression of YKT6 was closely associated with prognosis of HCC patients.^[11]^ Recent research about oral squamous cell carcinoma (OSCC) suggested that overexpression of YKT6 was related to cell invasion and metastasis, and low level of YKT6 was associated with CD8+ T cell infiltration.^[12]^ Pancreatic cancer studies also found that YKT6 was significantly upregulated in pancreatic cancer cells.^[13–15]^ However, studies of YKT6 have been confined to a few types of tumors, and its role in other tumors remains elusive.

In the study, we used TGCA project and GEO databases to investigate expression profiles of YKT6 across different types of cancer in a pan-cancer analysis. A number of factors, including gene expression, survival prognosis, genetic alteration, DNA methylation, immune infiltration, and gene enrichment analysis, were considered to explore the potential molecular mechanism by which YKT6 was involved in the pathogenesis or clinical prognosis of cancer.

## 2. Materials and methods

### 2.1. Gene expression analysis

The tumor immune estimation resource, vision 2 (TIMER2, web: http://timer.cistrome.org/) was used to analyze the differential expression of YKT6 between tumor and normal tissues in TCGA tumors. Gene Expression Profiling Interactive Analysis, version 2 (GEPIA2, web: http://gepia2.cancer-pku.cn/#analysis) was used to obtain box plots of Genotype-Tissue Expression (GTEx) database. The log_2_FC (fold change) cutoff was set 1, and a *P* value cutoff was .01. GEPIA2 tool was used to acquire the violin plots of YKT6 expression in different types of pathological stages of TCGA tumors. The UALCAN online toll (https://ualcan.path.uab.edu/analysis.html) was used to analyze cancer Omics data.^[16]^ The protein expression analysis dataset was got from clinical proteomic tumor analysis consortium (*CPTAC*) dataset by using UCLCAN.

### 2.2. Survival prognosis analysis

Gene Expression Profiling Interactive Analysis, version 2 (GEPIA2, web: http://gepia2.cancer-pku.cn/#analysis) was used to analyze overall survival (OS) and disease-free survival (DFS) significance map data (setting Group cutoff = median) of YKT6 gene in all TCGA tumors. Log-Rank method was used for statistical hypothesis test.

### 2.3. Genetic alteration analysis

The cBioPortal tool (http://www.cbioportal.org/) was used to obtain genetic alteration characteristics of YKT6.^[17]^ Genetic alteration characteristics including alteration frequency, mutation type, mutated site information, copy number alteration and three-dimensional (3D) structure of protein were collected. Survival data including overall, progression-free, disease-free and disease-free survival were obtained in TCGA tumors with or without YKT6 genetic alteration with “Comparison.” We went further detecting the relationship between YKT6 and tumor mutational burden (TMB) and microsatellite instability (MSI) with tumors in TCGA by using R package and R language software.

### 2.4. YKT6 methylation analysis

The level of methylation of YKT6 in different tumors was obtained from TCGA dataset by using UCLCAN.^[16]^ And multiple probes of YKT6 associated with DNA methylation in diverse types of tumor of TCGA were detected by the method of R package and R language software.

### 2.5. Immune infiltration analysis

We then used TIMER2 online tool to acquire the data of association between YKT6 level and immune infiltration. The gene of YKT6 was entered into “gene name field” for analysis. The results of the selected immune cells were performed by the method of a heatmap and scatter plots. *The TIMER algorithms, EPIC, XCELL, MCPCOUNTER, CIBERSORT*-*ABS, QUANTISEQ and CIBERSORT* were used to evaluate the degree of immune infiltrating situation.^[18]^

### 2.6. YKT6-related gene enrichment analysis

The STRING online tool (https://cn.string-db.org/) was used to detect the proteins which bind to YKT6. The following main parameters were set: score for minimum interaction required [“low confidence (0.150)”], maximum number of interactors shown (“no more than 50”), examples of network edges (“evidence”) and active interactions (“experiments”). Finally, the available experimentally determined YKT6-binding proteins were detected.

Based on the datasets of all TCGA tumors and normal tissues, we identified the top 100 YKT6-correlated targeting genes by using GEPIA2 platform. Analysis of gene Pearson correlation pairwise was conducted between YKT6 and genes which were selected. The mean of log2 TPM was used to calculate the dot plot. The heatmap data was presented using TIMER2^’^s “Gene_Corr” module. Spearman’s rank correlation was calculated using purity-adjusted test.

The Kyoto Encyclopedia of Genes and Genomes (KEGG) pathway were analyzed by using DAVID website and the “ggplot2” R packages. The results of KEGG were shown as bubble chart. Finally, the gene ontology (GO) enrichment was analyzed by the method of “cluster Profiler” R package and R language software. As a results of GO, bar plots were produced.

## 3. Results

### 3.1. Gene expression analysis data

**Figure 1.**
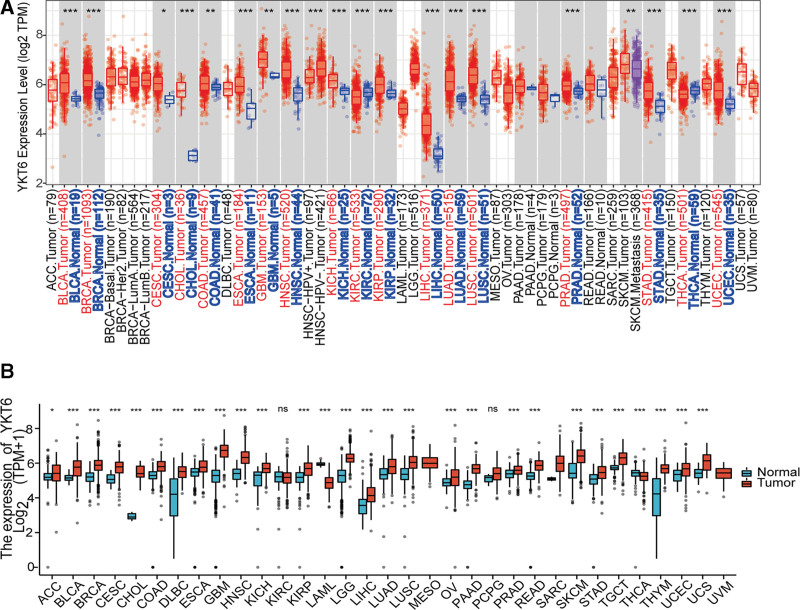
Expression level of YKT6 gene in different types of human cancers (A) expression of YKT6 in different types of cancers from TCGA was analyzed by TIMER2 (*P < .05,**P < .01,***P < .001). (B) expression of YKT6 was analyzed by using TCGA and GTEx databases (*P < .05,**P < .01,***P < .001). ACC = adrenocortical cancer, BLCA = bladder urothelial carcinoma, BRCA = breast invasive carcinoma, CESC = cervical squamous cell carcinoma and endocervical adenocarcinoma, CHOL = cholangiocarcinoma, COAD = colon adenocarcinoma, DLBC = Lymphoid Neoplasm Diffuse Large B-cell, ESCA = esophageal carcinoma, GBM = glioblastoma multiforme, HNSC = head and neck squamous cell carcinoma, KICH = kidney chromophobe, KIRC = kidney renal clear cell carcinoma, KIRP = kidney papillary cell carcinoma, LAML = acute myeloid leukemia, LIHC = liver hepatocellular carcinoma, LGG = lower grade glioma, LUAD = lung adenocarcinoma, LUSC = lung squamous cell carcinoma, MESO = mesothelioma, OV = ovarian serous cystadenocarcinoma, PAAD = pancreatic adenocarcinoma, PCPG = pheochromocytoma & paraganglioma, PRAD = prostate adenocarcinoma, READ = rectum adenocarcinoma, SARC = sarcoma, SKCM = skin cutaneous melanoma, STAD = stomach adenocarcinoma, TCGA = the Cancer Genome Atlas, TGCT = testicular germ cell tumors, THYM = thymoma, THCA = thyroid carcinoma, UCEC = uterine corpus endometrial carcinoma, UCS = uterine carcinosarcoma, UVM = ocular melanomas.

TIMER2 was used to analyze the differential expression of YKT6 between tumor and normal tissues in TCGA tumors. As shown in Figure 1A, the expression of YKT6 in Bladder Urothelial Carcinoma (BLCA), Breast invasive carcinoma (BRCA), Cholangiocarcinoma (CHOL), Esophageal carcinoma (ESCA), Head and Neck squamous cell carcinoma (HNSC), Kidney Chromophobe (KICH), Kidney renal papillary cell carcinoma (KIRP), Liver hepatocellular carcinoma (LIHC), Lung adenocarcinoma (LUAD), Lung squamous cell carcinoma (LUSC), Prostate adenocarcinoma (PRAD), Stomach adenocarcinoma (STAD), Uterine Corpus Endometrial Carcinoma (UCEC) (*P* ˂ .001), colon adenocarcinoma (COAD), glioblastoma multiforme (GBM) (*P* ˂ .01), Cervical squamous cell carcinoma and endocervical adenocarcinoma (CESC) (*P* ˂ .05) was significantly overexpressed compared with the corresponding control tissues. However, the expression of YKT6 was significantly downregulated in Kidney renal clear cell carcinoma (KIRC) and Thyroid carcinoma (THCA) (*P* ˂ .001).

We went further to analyzing the expression of YKT6 by using tumor tissues and normal tissues in TCGA and GTEx data to get detailed statistical calculations. As shown in Figure 1B, YKT6 was significantly upregulated in Adrenocortical carcinoma (ACC) (*P* ˂ .05), BLCA, BRCA, CESC, CHOL, COAD, Lymphoid Neoplasm Diffuse Large B-cell (DLBC), ESCA, GBM, HNSC, KICH, KIRP, Brain Lower Grade Glioma (LGG), LIHC, LUAD, LUSC, Ovarian serous cystadenocarcinoma (OV), Pancreatic adenocarcinoma (PAAD), PRAD, Rectum adenocarcinoma (READ), Sarcoma (SARC), Skin Cutaneous Melanoma (SKCM), STAD, Testicular Germ Cell Tumors (TGCT), Thymoma (THYM), UCEC and Uterine Carcinosarcoma (UCS) (*P* ˂ .001). By contrast, the expression of YKT6 was low expressed in Acute Myeloid Leukemia (LAML) and THCA (*P* ˂ .001). There were no statistical differences in KIRC and Pheochromocytoma and Paraganglioma (PCPG).

**Figure 2.**
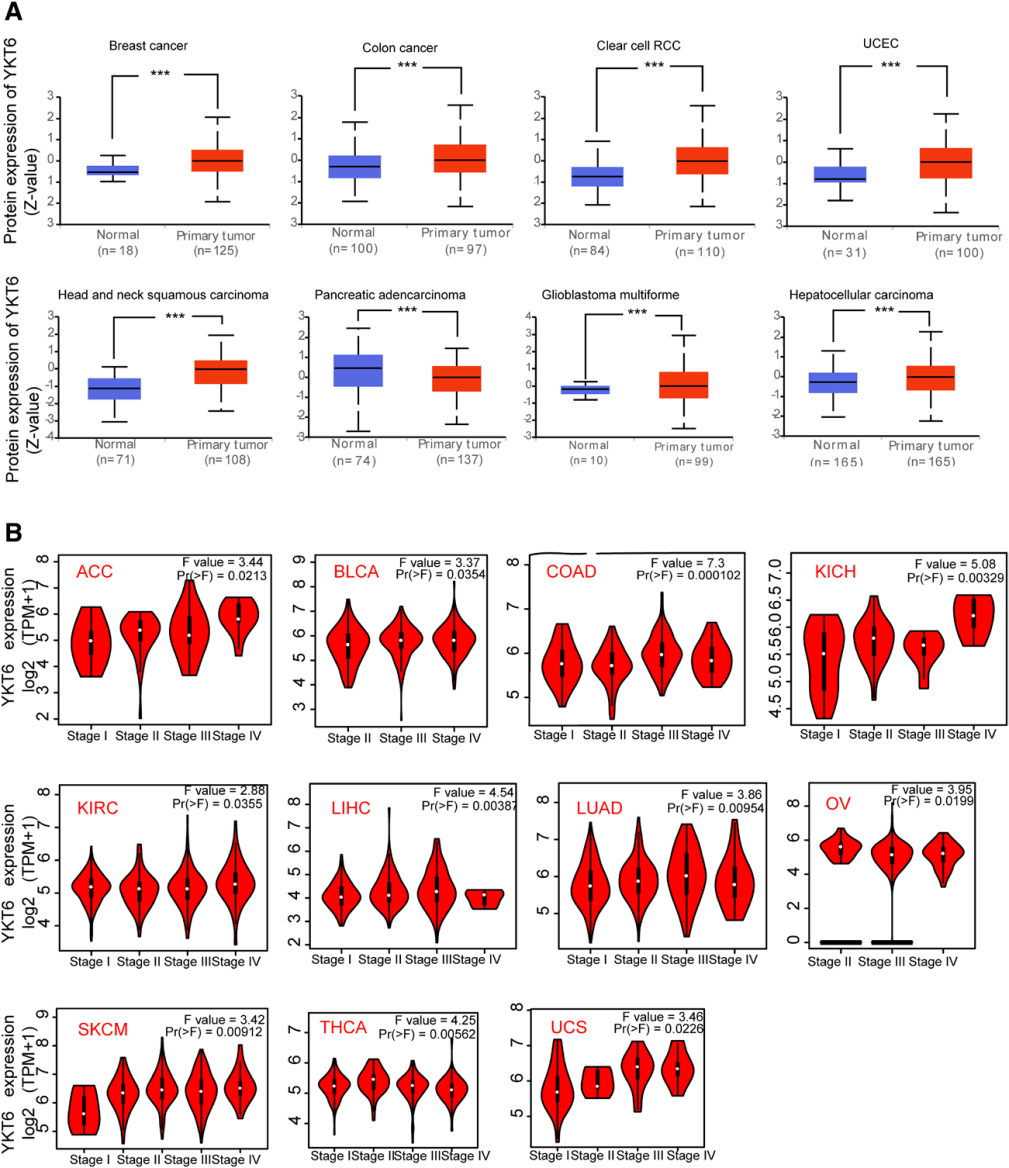
Expression level of YKT6 gene in different tumors and pathological stages. (A) Based on the CPTAC dataset, the level of YKT6 total protein was analyzed between normal tissue and primary tissue of breast cancer, colon cancer, clear cell RCC, UCEC, HNSC, PAAD, GBM, and HCC. (***P < .001). (B) Based on the TCGA data, the expression of the YKT6 was analyzed by the main pathological stages (stage I, stage II, stage III, and stage IV) of ACC, BLCA, COAD, KICH, KIRC, LIHC, LUAD, OV, SKCM, THCA and UCS. Log2 (TPM + 1) was applied for log-scale. ACC = adrenocortical cancer, BLCA = bladder urothelial carcinoma, COAD = colon adenocarcinoma, KICH = kidney chromophobe, KIRC = kidney renal clear cell carcinoma, LIHC = liver hepatocellular carcinoma, LUAD = lung adenocarcinoma, OV = ovarian serous cystadenocarcinoma, SKCM = skin cutaneous melanoma, THCA = thyroid carcinoma, UCS = uterine carcinosarcoma.

We then analyzed the YKT6 protein level in different tumors of TCGA by using CPTAC dataset. As shown in Figure 2A, protein expression of YKT was significantly overexpressed in BRCA, COAD, GBM, HNSC, KIRC, LIHC, UCEC, and PAAD (*P* ˂ .001) compared with corresponding normal tissues.

Furthermore, we use GEPIA2 online tool to detect expression of YKT6 in different tumor stages. The data of Figure 2B showed that there was a significant relation between level of YKT6 and the pathological stages of several tumors, including ACC (*P* = .0213), BLCA (*P* = .0354), COAD (*P* = .0000102), KICH (*P* = .000329), KIRC (*P* = .0355), LIHC (*P* = .00387), LUAD (*P* = .00954), OV (*P* = .0199), SKCM (*P* = .00912), THCA (*P* = .00562), UCS (*P* = .0226).

### 3.2. Survival prognosis analysis

**Figure 3.**
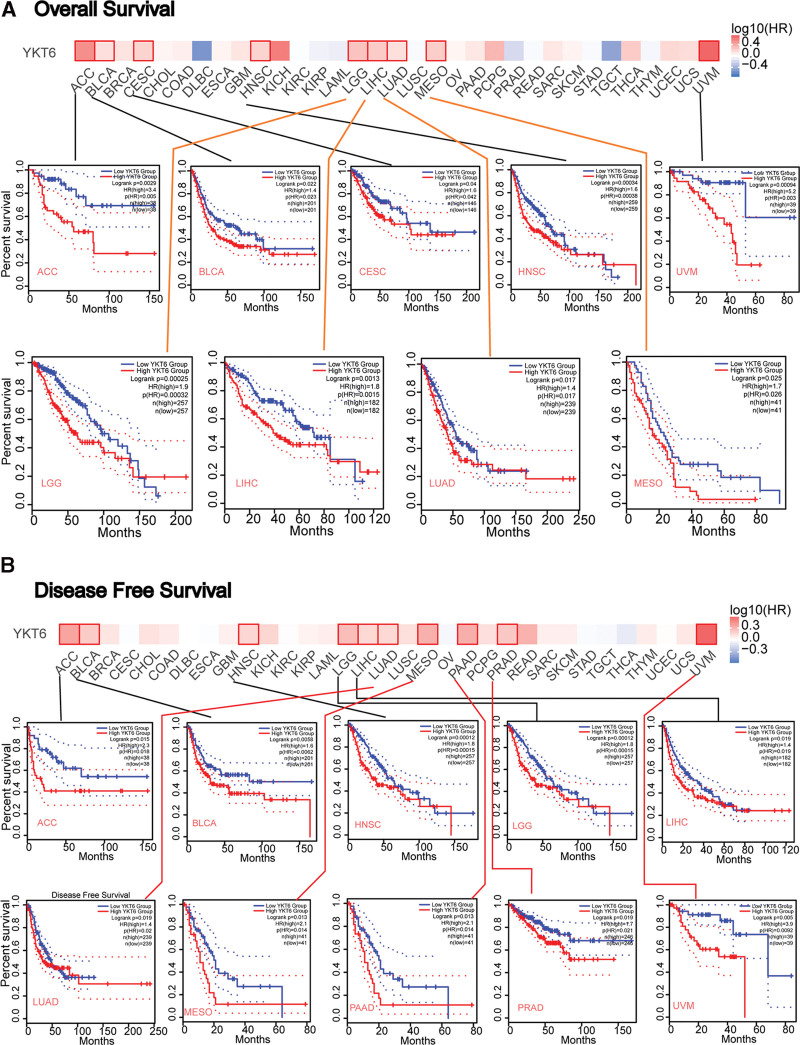
The correlation between YKT6 gene expression and survival prognosis of cancers in TCGA. We used the GEPIA2 tool to perform overall survival (A) and diseasefree survival (B) analyses of different tumors in TCGA by YKT6 gene expression. The survival map and Kaplan–Meier curves with positive results are shown. ACC = adrenocortical cancer, BLCA = bladder urothelial carcinoma, BRCA = breast invasive carcinoma, CESC = cervical squamous cell carcinoma and endocervical adenocarcinoma, CHOL = cholangiocarcinoma, COAD = colon adenocarcinoma, DLBC = Lymphoid Neoplasm Diffuse Large B-cell, ESCA = esophageal carcinoma, GBM = glioblastoma multiforme, HNSC = head and neck squamous cell carcinoma, KICH = kidney chromophobe, KIRC = kidney renal clear cell carcinoma, KIRP = kidney papillary cell carcinoma, LAML = acute myeloid leukemia, LIHC = liver hepatocellular carcinoma, LGG = lower grade glioma, LUAD = lung adenocarcinoma, LUSC = lung squamous cell carcinoma, MESO = mesothelioma, OV = ovarian serous cystadenocarcinoma, PAAD = pancreatic adenocarcinoma, PCPG = pheochromocytoma & paraganglioma, PRAD = prostate adenocarcinoma, READ = rectum adenocarcinoma, SARC = sarcoma, SKCM = skin cutaneous melanoma, STAD = stomach adenocarcinoma, TCGA = the Cancer Genome Atlas, TGCT = testicular germ cell tumors, THYM = thymoma, THCA = thyroid carcinoma, UCEC = uterine corpus endometrial carcinoma, UCS = uterine carcinosarcoma, UVM = ocular melanomas.

To evaluate the relationship between YKT6 and the prognosis of patients with diverse kinds of cancer, the tumors were dichotomized into 2 groups (high-expression and low-expression groups) based on the level of YKT6 in TCGA and GEO datasets. As data displayed in Figure 3A, we found that high-expression of YKT6 was positively correlated with poor prognosis of OS in different types of tumors including ACC (*P* = .0029), BLCA (*P* = .022), CESE (*P* = .04), Head and Neck squamous cell carcinoma (HNSC, *P* = .00034), LGG (*P* = .00025), LIHC (*P* = .0013), LUAD (*P* = .017), MESO (*P* = .025), and UVM (*P* = .00094). As shown in Figure 3B, high-expression of YKT6 was associated with poor DFS prognosis for ACC (*P* = .015), BLCA (*P* = .0058), HNSC (*P* = .022), LGG (*P* = .00012), LIHC (*P* = .019), LUAD (*P* = .019), MESO (*P* = .013), PAAD (*P* = .0014), PRAD (*P* = .019), and UVM (*P* = .005).

### 3.3. Genetic alteration analysis

**Figure 4.**
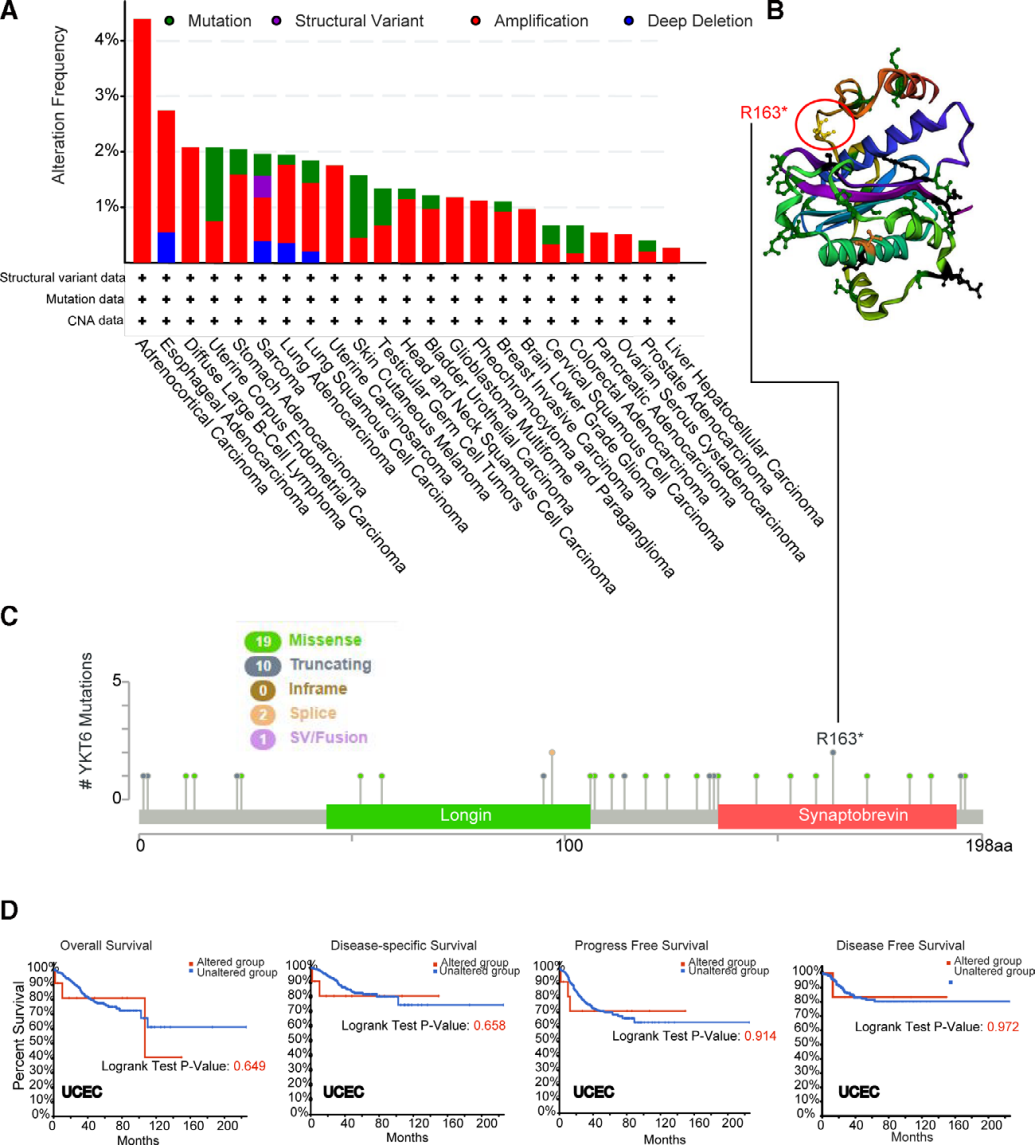
Mutation feature of YK6 in different types of cancers of TCGA. The mutation features of YKT6 were analyzed by using the cBioPortal tool. (A) The alteration frequency of mutation type was displayed. (B) We also display the mutation site with the highest alteration frequency (R163*) in the 3D structure of YKT6. (C) The alteration frequency of mutation site was displayed. (D) The potential correlation between mutation status and overall, disease-specific, disease-free and progression-free survival of UCEC were analyzed by using the cBioPortal tool. TCGA = the Cancer Genome Atlas, UCEC = uterine corpus endometrial carcinoma.

The genetic alteration of YKT6 in different types of tumors from TCGA was analyzed. As data described in Figure 4A, YKT6 alteration frequency (>4%) is the highest in ACC, with the primary alteration type being “Amplification.” We obtained that the second-most frequency of YKT6 alteration (>1.5%) in cases with ESCA with “Amplification” as the main type. “Amplification” was the sole form of variation in DLBC, UCS, GBM, and PCPG. The additional mutations and location of YKT6 were shown on Figure 4B. No predominant genetic alterations were obtained. Locations of genetic alterations appeared to be sporadic. For example, a truncating mutation, R163^*^ alteration, within the Synaptobrevin domain, was only found in 2 patients with UCEC (Fig. 4C). In UCEC patients, we detected whether YKT6 genetic alterations affect clinical survival prognosis. We found that prognosis in terms of OS (*P* = .495), DFS (*P* = .304), progression-free survival (PFS) (*P* = .125), and disease-specific (DS) (*P* = .268) were no significant difference between YKT6 altered group and unaltered group (Fig. 4D).

**Figure 5.**
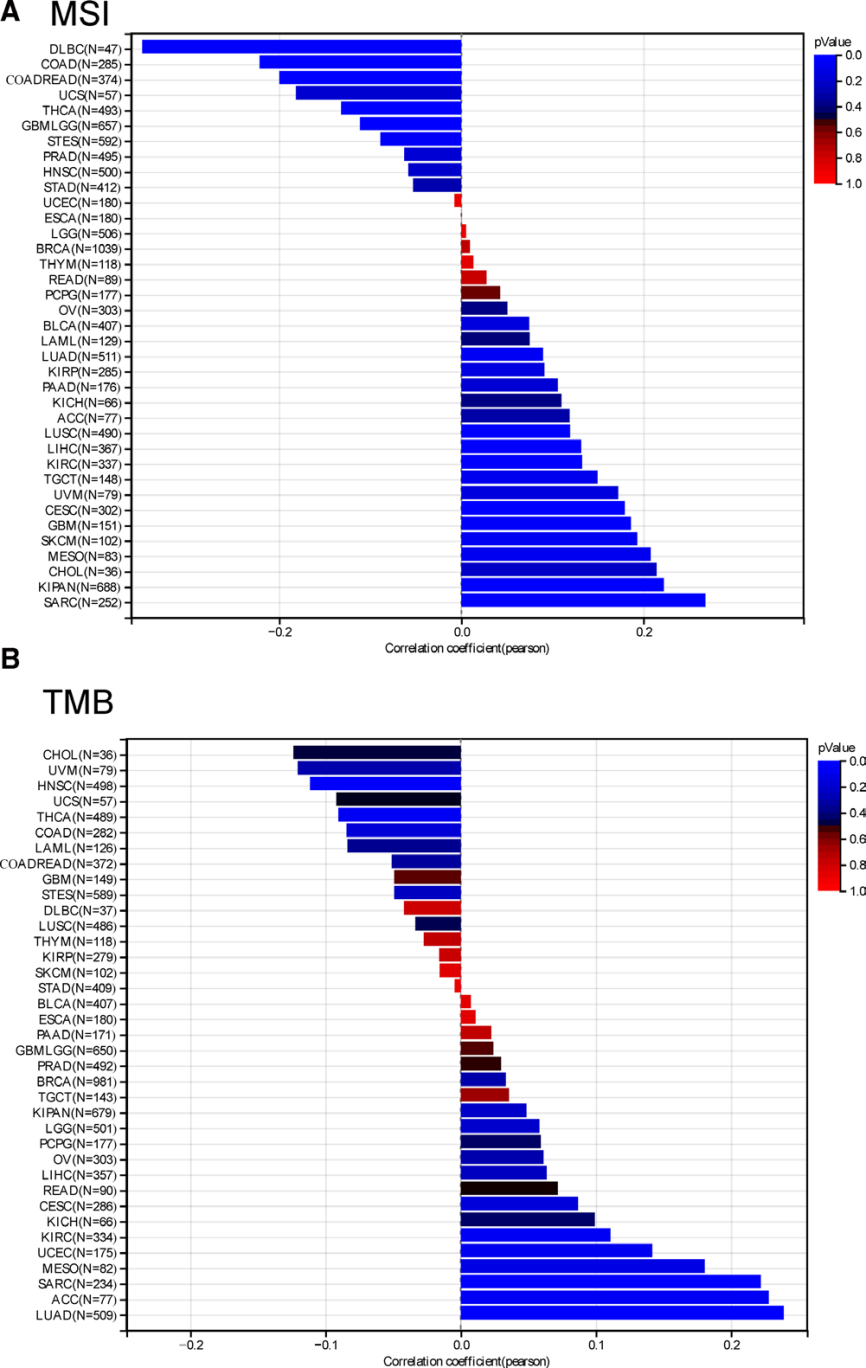
The correlation of YKY6 expression and TMB, MSI in cancers. ACC = adrenocortical cancer, BLCA = bladder urothelial carcinoma, BRCA = breast invasive carcinoma, CESC = cervical squamous cell carcinoma and endocervical adenocarcinoma, CHOL = cholangiocarcinoma, COAD = colon adenocarcinoma, DLBC = Lymphoid Neoplasm Diffuse Large B-cell, ESCA = esophageal carcinoma, GBM = glioblastoma multiforme, HNSC = head and neck squamous cell carcinoma, KICH = kidney chromophobe, KIRC = kidney renal clear cell carcinoma, KIRP = kidney papillary cell carcinoma, LAML = acute myeloid leukemia, LIHC = liver hepatocellular carcinoma, LGG = lower grade glioma, LUAD = lung adenocarcinoma, LUSC = lung squamous cell carcinoma, MESO = mesothelioma, MSI = microsatellite instability, OV = ovarian serous cystadenocarcinoma, PAAD = pancreatic adenocarcinoma, PCPG = pheochromocytoma & paraganglioma, PRAD = prostate adenocarcinoma, READ = rectum adenocarcinoma, SARC = sarcoma, SKCM = skin cutaneous melanoma, STAD = stomach adenocarcinoma, TCGA = the Cancer Genome Atlas, TGCT = testicular germ cell tumors, THYM = thymoma, THCA = thyroid carcinoma, TMB = tumor mutational burden, UCEC = uterine corpus endometrial carcinoma, UCS = uterine carcinosarcoma, UVM = ocular melanomas.

Moreover, we detected the relationship between YKT6 and TMB and MSI with tumors by using TCGA. As described in Figure 5A, we obtained that the expression of YKT6 was positively associated with TMB in LUAD (*P* = 4.92e-8), SARC (*P* = .00062), KIRC (*P* = .043) and ACC (*P* = .046). While, the expression of YKT6 was negative association with TMB in HNSC (*P* = .013) and THCA (*P* = .046). As described in Figure 5B, we also explored that YKT6 expression was positively correlated with MSI in GBM (*P* = .022), CESC (*P* = .0017), LUAD (*P* = .043), SARC (*P* = .000016), KIPAN (*P* = 3.69e-9), KIRC (*P* = .015), LUSC (*P* = .0082), and LIHC (*P* = .012). By contrast, the expression of YKT6 was negatively correlated with MSI in GBMLGG (*P* = .0044), COAD (*P* = .00017), COADREAD (*P* = .00010), STES (*P* = .031), THCA (*P* = .0033), and DLBC (*P* = .016). These results suggested the genetic alteration of YKT6 may be considered as potential novel drivers of some tumors.

### 3.4. DNA methylation analysis

**Figure 6.**
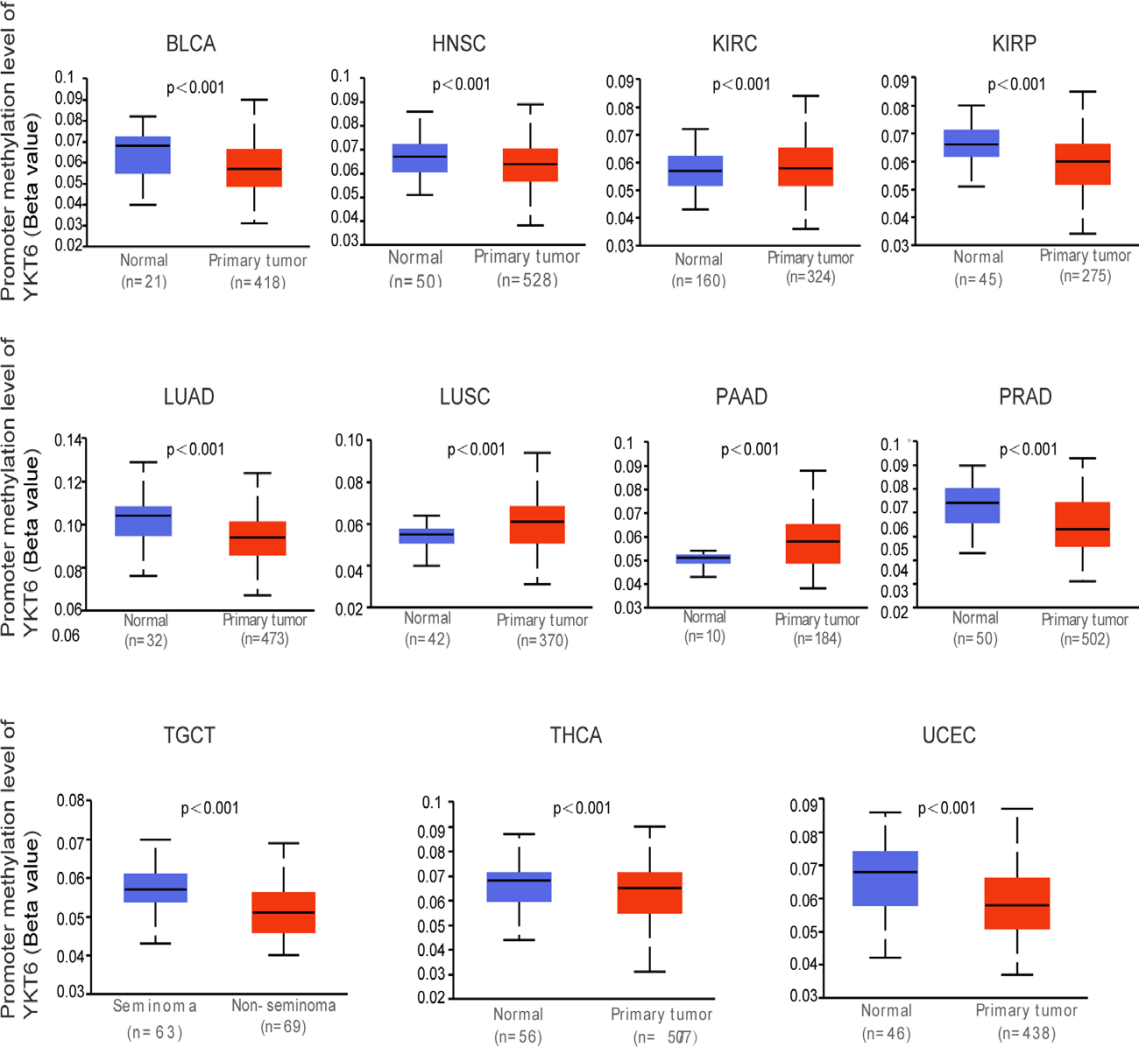
The level of methylation of YKT6 in different tumors was obtained from TCGA dataset by using UCLCAN. BLCA = bladder urothelial carcinoma, HNSC = head and neck squamous cell carcinoma, KIRC = kidney renal clear cell carcinoma, KIRP = kidney papillary cell carcinoma, LUAD = lung adenocarcinoma, LUSC = lung squamous cell carcinoma, PAAD = pancreatic adenocarcinoma, PRAD = prostate adenocarcinoma, TCGA = the Cancer Genome Atlas, TGCT = testicular germ cell tumors, THCA = thyroid carcinoma, UCEC = uterine corpus endometrial carcinoma.

DNA methylation, as an important epigenetic regulator of postreplication, played a significant role in tumorigenesis.^[19]^ Figure 6 showed that there was a hypermethylation status in promoter region of YKT6 in KIRC, LUSC, and PAAD. While, there was a hypomethylation level in the promoter region of YKT6 in BLCA, HNSC, KIRP, LUAD, PRAD, TGCT, THCA, and UCEC. The occurrence and development of tumors was affected by up-regulated or down-regulated DNA methylation state of target gene.

**Figure 7.**
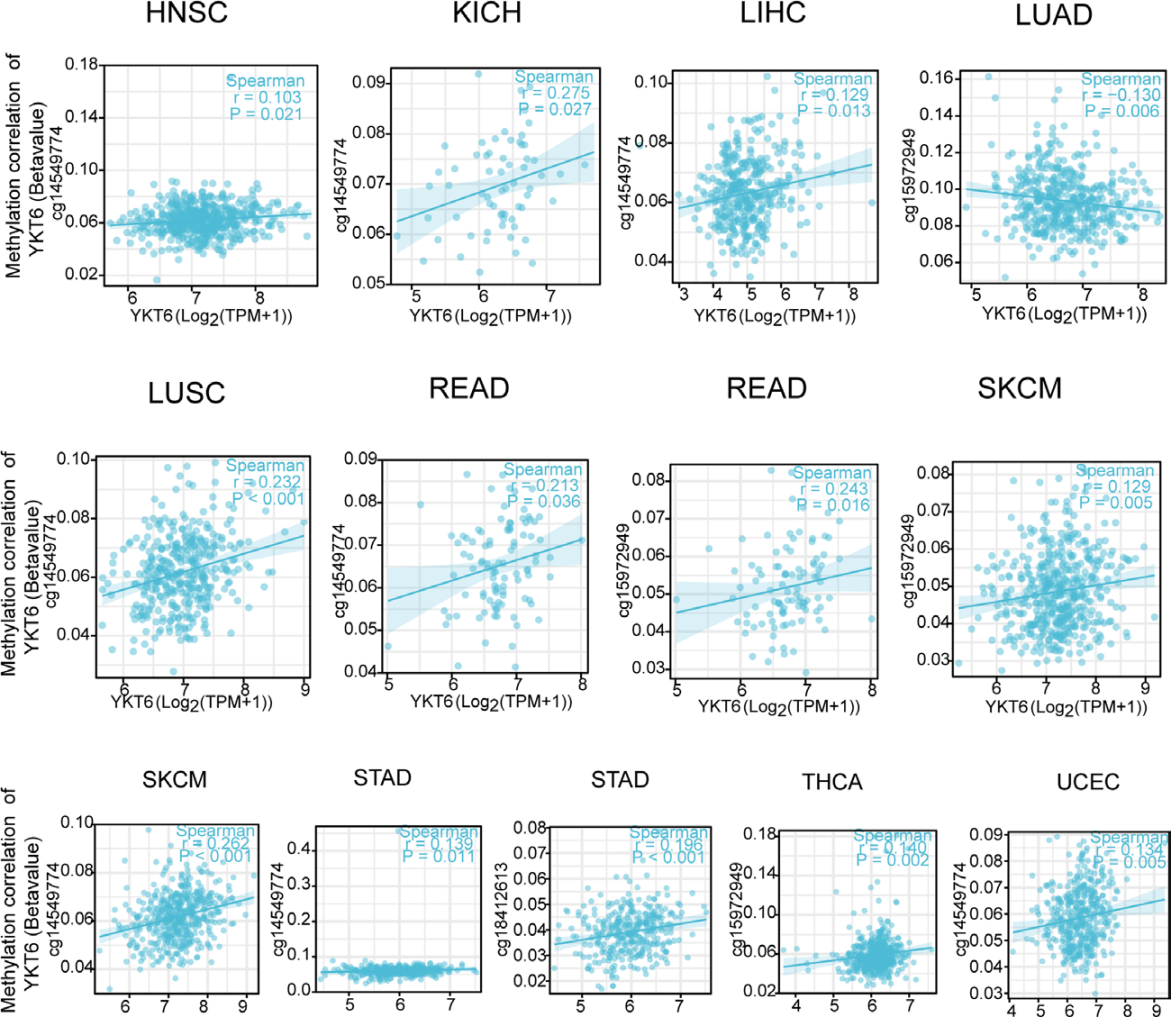
Multiple probes of YKT6 associated with DNA methylation in diverse types of tumor of TCGA were detected by the method of R package and R language software. HNSC = head and neck squamous cell carcinoma, KICH = kidney chromophobe, LIHC = liver hepatocellular carcinoma, LUAD = lung adenocarcinoma, LUSC = lung squamous cell carcinoma, READ = rectum adenocarcinoma, SKCM = skin cutaneous melanoma, STAD = stomach adenocarcinoma, TCGA = the Cancer Genome Atlas, THCA = thyroid carcinoma, UCEC = uterine corpus endometrial carcinoma.

We also used “R packages” to explore the relationship between YKT6 DNA methylation and etiopathogenesis of diverse types of tumors in TCGA database. As the data shown in Figure 7, we obtained a significant positive association of YKT6 DNA methylation and gene expression at the probe of the non-promoter region as cg14549774 in several tumors. We also explored that YKT6 DNA methylation was positively related with cg15972849 in READ, SKCM, and THCA. Meanwhile, YKT6 DNA methylation was negatively correlated with cg15972849 in RRCA, GBMLGG, and LUAD. Moreover, we detected that YKT6 DNA methylation was positively associated with cg1841261 in STAD.

### 3.5. Immune infiltration analysis

**Figure 8.**
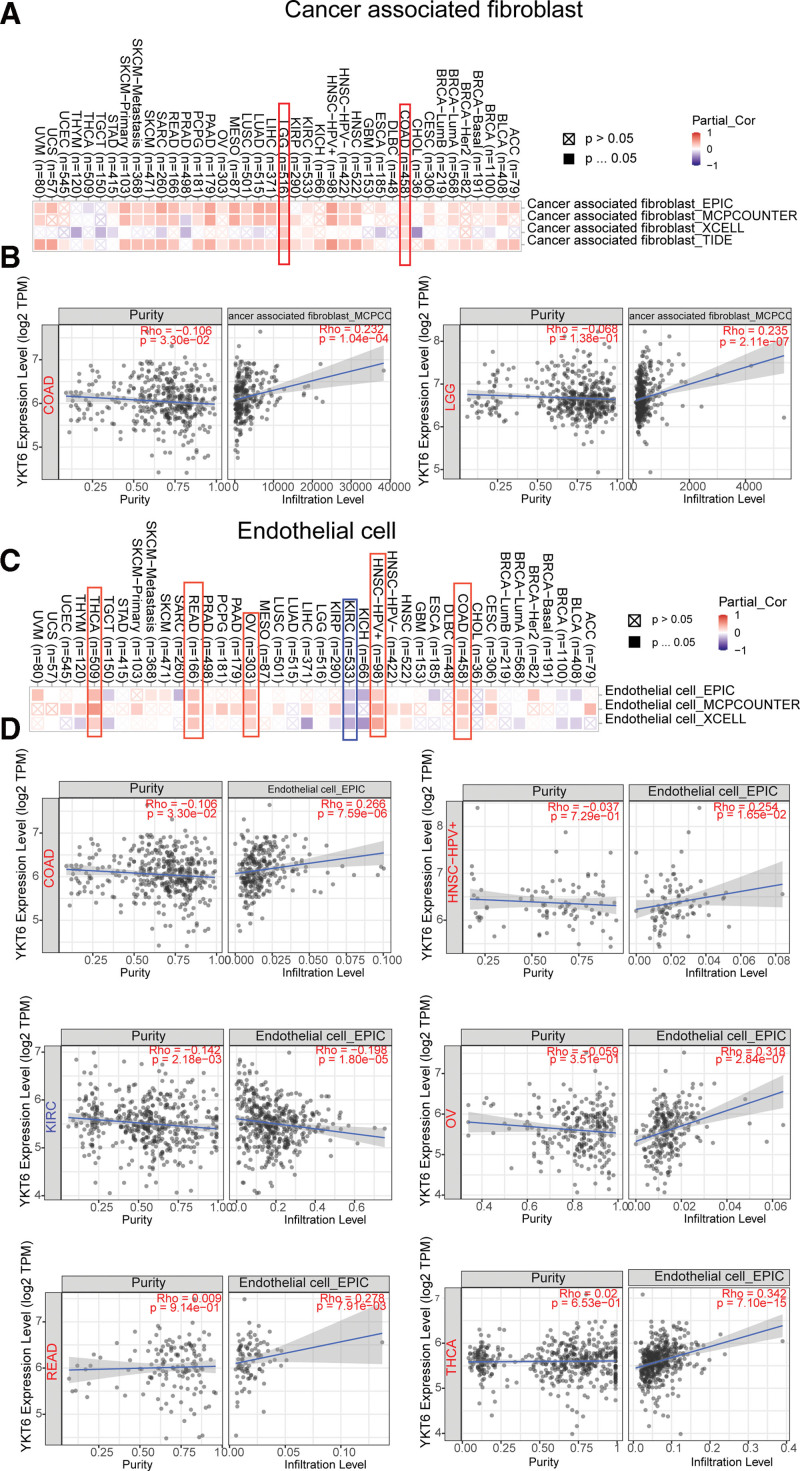
Correlation analysis between YKT6 expression and immune infiltration of cancer-associated fibroblasts (A), and endothelial cell (C). Different algorithms were used to explore the potential correlation between the expression level of the YKT6 gene and the infiltration level of cancer-associated fibroblasts across all types of cancer in TCGA (B). Different algorithms were used to explore the potential correlation between the expression level of the YKT6 gene and the infiltration level of endothelial cell across all types of cancer in TCGA (D). ACC = adrenocortical cancer, BLCA = bladder urothelial carcinoma, BRCA = breast invasive carcinoma, CESC = cervical squamous cell carcinoma and endocervical adenocarcinoma, CHOL = cholangiocarcinoma, COAD = colon adenocarcinoma, DLBC = Lymphoid Neoplasm Diffuse Large B-cell, ESCA = esophageal carcinoma, GBM = glioblastoma multiforme, HNSC = head and neck squamous cell carcinoma, KICH = kidney chromophobe, KIRC = kidney renal clear cell carcinoma, KIRP = kidney papillary cell carcinoma, LAML = acute myeloid leukemia, LIHC = liver hepatocellular carcinoma, LGG = lower grade glioma, LUAD = lung adenocarcinoma, LUSC = lung squamous cell carcinoma, MESO = mesothelioma, OV = ovarian serous cystadenocarcinoma, PAAD = pancreatic adenocarcinoma, PCPG = pheochromocytoma & paraganglioma, PRAD = prostate adenocarcinoma, READ = rectum adenocarcinoma, SARC = sarcoma, SKCM = skin cutaneous melanoma, STAD = stomach adenocarcinoma, TCGA = the Cancer Genome Atlas, TGCT = testicular germ cell tumors, THYM = thymoma, THCA = thyroid carcinoma, UCEC = uterine corpus endometrial carcinoma, UCS = uterine carcinosarcoma, UVM = ocular melanomas.

Tumor-infiltrating immune cells, as an integral part of tumor microenvironment (TME), played a crucial role in tumor progression and development.^[20,21]^ We then used the TIMER, CIBERSORT, CIBERSORT-ABS, TIDE, XCEL, MCPCOUNTER, QUANTISEQ, and EPIC algorithms to detect the possible association between the different immune infiltration and immune cells and YKT6 level in different types of tumors in TCGA. We detected that YKT6 level was positively associated with cancer-associated fibroblasts (CAFs) for TCGA tumors of COAD and LGG (Fig. 8A and B).

Furthermore, as data shown in Figure 8C and D, we discovered a significantly positively association between YKT6 level and endothelial cell in tumors of COAD, HNSC-HPV+, OV, READ, and THCA. While a negative relationship was obtained between YKT6 expression and endothelial cell in KIRC.

### 3.6. Enrichment analysis of YKT6-related gene

**Figure 9.**
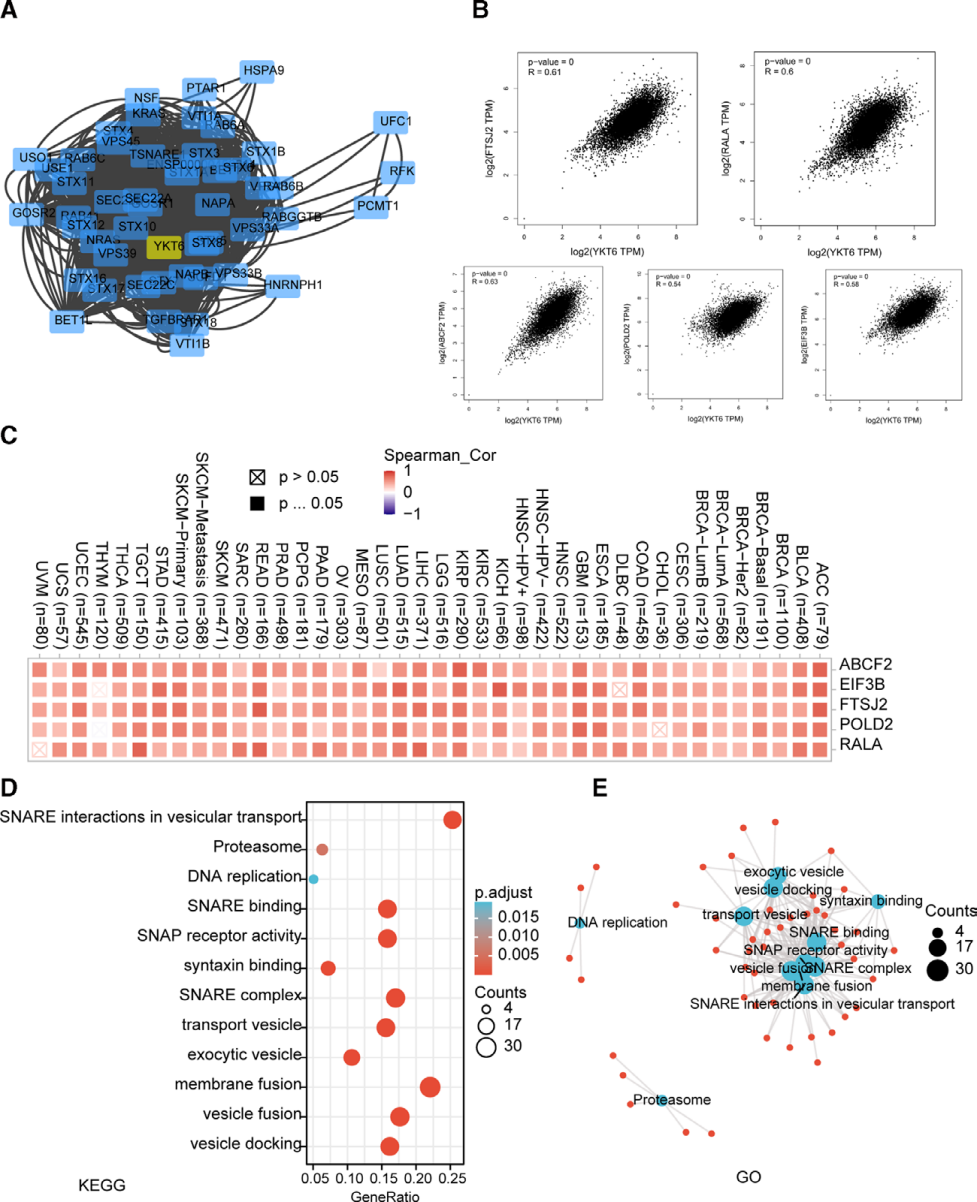
YKT6-related gene enrichment analysis. (A) We first obtained the available experimentally determined YKT6-binding proteins using the STRING tool. (B) Using the GEPIA2 approach, we also obtained the top 100 YKT6-correlated genes in TCGA projects and analyzed the expression correlation between YKT6 and selected targeting genes including ABCF2, EIF3B, FTSJ2, POLD2, and RALA. (C) The corresponding heatmap data in the detailed cancer types are displayed. (D) Based on the YKT6-binding and interacted genes, KEGG pathway analysis was performed. (E) GO enrichment analysis of YKT6-binding and interacted genes in molecular is also shown. ACC = adrenocortical cancer, BLCA = bladder urothelial carcinoma, BRCA = breast invasive carcinoma, CESC = cervical squamous cell carcinoma and endocervical adenocarcinoma, CHOL = cholangiocarcinoma, COAD = colon adenocarcinoma, DLBC = Lymphoid Neoplasm Diffuse Large B-cell, ESCA = esophageal carcinoma, GBM = glioblastoma multiforme, GO = gene ontology, HNSC = head and neck squamous cell carcinoma, KEGG = Kyoto Encyclopedia of Genes and Genomes, KICH = kidney chromophobe, KIRC = kidney renal clear cell carcinoma, KIRP = kidney papillary cell carcinoma, LAML = acute myeloid leukemia, LIHC = liver hepatocellular carcinoma, LGG = lower grade glioma, LUAD = lung adenocarcinoma, LUSC = lung squamous cell carcinoma, MESO = mesothelioma, OV = ovarian serous cystadenocarcinoma, PAAD = pancreatic adenocarcinoma, PCPG = pheochromocytoma & paraganglioma, PRAD = prostate adenocarcinoma, READ = rectum adenocarcinoma, SARC = sarcoma, SKCM = skin cutaneous melanoma, STAD = stomach adenocarcinoma, TCGA = the Cancer Genome Atlas, TGCT = testicular germ cell tumors, THYM = thymoma, THCA = thyroid carcinoma, UCEC = uterine corpus endometrial carcinoma, UCS = uterine carcinosarcoma, UVM = ocular melanomas.

To explore the mechanism of YKT6 in tumorigenesis, we then sought to identify YKT6-binding proteins and YKT6 level-correlated genes for a variety of pathway enrichment analyses. In Figure 9A, we got 50 YKT6-binding proteins by using STRING online tool. We obtained the top 100 genes which associated with YKT6 expression by using GEPIA2 online tool. As data shown in Figure 9B, we found that the expression of YKT6 was positively related with FTSJ2 (*R* = 0.61), RALA (*R* = 0.61), ABCF2 (*R* = 0.63), POLD2 (*R* = 0.54) and EIF3B (*R* = 0.58) genes (all *P < .001*). Heatmap data showed that YKT6 gene had a significant positive association with the above 5 genes in the majority of tumors (Fig. 9C).

And then, we used KEGG pathway and GO enrichment analyses to explore the functions of YKT6. We found that “Syntaxin binding,” “SNARE complex,” “vesicle fusion” and “DNA replication” may be involved in the influence of YKT6 on tumor pathogenesis (Fig. 9D and E).

## 4. Discussion

Previous researches have shown that YKT6 has emerged as a critical protein implicated in multitudes of trafficking events.^[2,22]^ Recently studies have also revealed that YKT6 has been involved in the progression of several cancers, including NSCLC, HCC, PAAD, BRCA, and OSCC.^[8–13]^ It remains to be answered that whether YKT6 can play an important role in the progression of different types of cancers via certain common molecular mechanisms.

In our study, YKT6 was significantly elevated in majority of tumors through TCGA and GEO databases, which is not in accordance with the altered protein levels in KIRC in the CPTAC dataset. This suggested that over-expressed RNA expressions of YKT6 may be usual, but it could not reflect actual protein level or response to certain types of cancer. In addition, we detected that upregulation of YKT6 commonly predicted poor OS and DFS for patients with tumors expressing over-expression of YKT6, such as ACC, BLCA, HNSC, LGG, LIHC, and so on. These findings reveal that YKT6 is a potential biomarker for the prognosis of patients with tumors. Yang et al^[9]^ recently found that the high level of YKT6 related with worse prognoses in patients with OSCC which is the most common subtype of Head and Neck squamous cell carcinoma (HNSC). Similarly, we found that high YKT6 expression was related to poor OS (*P* = .00034) and DFS (*P* = .022) in HNSC, which is in agreement with Yang’s recently report. YKT6 was also reported to have a detrimental effect on survival for NSCLC patients. Consistent with the finding, our results revealed a relation between high level of YKT6 and poor OS (*P* = .017) and DFS (*P* = .019) in patients with LUAD. Xu et al^[11]^ reported that over-expression of YTK6 was correlated with worse prognosis in patients with hepatocellular carcinoma. Similarly, we observed that high level of YKT6 was positive association with poor prognosis OS (*P* = .0013) and DFS (*P* = .019) in LIHC, which is in accordance with Xu’s report. Consequently, KT6 can play an important role in clinical prognosis of patients with tumors based on the clinical big data evidence. Many studies have shown that clinical survival prognosis was often associated with genetic alterations related to cancer progression.^[23,24]^ While, there is no potential relationship between YKT6 genetic alteration and clinical survival prognosis including OS (*P* = .495), DFS (*P* = .304), PFS (*P* = .125), and DS (*P* = .268) in UCEC patients.

Recently, researchers have become increasingly interested in exploring how YKT6 played a role in tumor. Throughout the genome, microsatellites are short stretches of DNA that are repeated, and MSI occurs when one or more repeats are added or removed.^[25]^ TMB is the total amount of mutations per DNA megabase.^[26]^Both TMB and MSI are 2 new biomarkers correlated with immunotherapy responses.^[27]^ In our present study, we firstly demonstrated the potential relationship between the level of YKT6 and TMB or MSI. We detected the significant relationship between YKT6 of several tumors and TMB and MSI with tumors in TCGA. Furthermore, we conducted a series of enrichment analyses using information on YKT6-binding components and YKT6 level-related genes through all tumors. We obtained the possible impact of “Syntaxin binding,” “SNARE complex,” “vesicle fusion” and “DNA replication” in the tumorigensis. Our study showed that YKT6 expression was positively related to cancer-associated fibroblasts for TCGA tumors of COAD and LGG. Furthermore, our findings demonstrated a significantly positively association between expression of YKT6 and endothelial cell in tumors of COAD, HNSC-HPV+, OV, READ and THCA. Meanwhile, a statistically negative relationship was obtained between YKT6 expression and endothelial cell in KIRC.

Methylation of DNA plays a crucial role in tumor development. Up-or down-regulation of DNA methylation levels can influence the expression of tumor gene, which then affects tumorigenesis and development.^[28,29]^ We found a potential association between DNA methylation and YKT6 gene. The difference of YKT6 methylation between tumor tissues and matched normal tissues has distinct results at different methylation sites. There remains a need for further evidence of how YKT6 DNA methylation might contribute to tumorigenesis.

In summary, our pan-cancer analysis of YKT6 revealed statistical association of YKT6 level with survival prognosis, genetic alteration, DNA methylation and immune infiltration across most tumors, which can contribute to understanding the role of YKT6 in tumoeigenesis from perspective of clinical tumor samples.

## Author contributions

**Conceptualization:** Xuezhong Zhang.

**Data curation:** Xuezhong Zhang, Mark Lloyd G. Dapar, Xin Zhang, Yingjun Chen.

**Methodology:** Xuezhong Zhang.

**Resources:** Xuezhong Zhang, Yingjun Chen.

**Software:** Xuezhong Zhang, Xin Zhang.

**Validation:** Mark Lloyd G. Dapar.

**Writing – original draft:** Xuezhong Zhang.

**Writing – review & editing:** Mark Lloyd G. Dapar.
